# Comparative clinical efficacy of percutaneous coaxial large-channel endoscopic lumbar interbody fusion and transforaminal lumbar interbody fusion for degenerative lumbar spinal stenosis: a retrospective study

**DOI:** 10.1186/s12891-024-07608-6

**Published:** 2024-06-26

**Authors:** Zige Liu, Tianxiang Yang, Jun Li, Desheng Chen

**Affiliations:** 1 School of Clinical Medicine, Guangxi Medical University, Nanning, 530000 China; 2 School of Clinical Medicine, Ningxia Medical University, Yinchuan, 750000 China; 3 Department of Orthopedic Surgery, People’s Hospital of Ningxia Hui Autonomous Region, Ningxia Medical University, Yinchuan, 750000 China

**Keywords:** Percutaneous coaxial large-channel endoscopic lumbar interbody fusion, Transforaminal lumbar interbody fusion, Degenerative lumbar spinal stenosis, Clinical efficacy, Retrospective study

## Abstract

This study aimed to evaluate the clinical efficacy of percutaneous coaxial large-channel endoscopic lumbar interbody fusion (PCLE-LIF) and transforaminal lumbar interbody fusion (TLIF) in the treatment of degenerative lumbar spinal stenosis. The clinical data of patients with degenerative lumbar spinal stenosis who underwent PCLE-LIF (experimental group) and TLIF (control group) surgery from September 2019 to September 2021 were retrospectively analyzed. We collected clinical data and compared the two groups in terms of perioperative parameters, treatment response rate, inflammatory response markers, postoperative complications, postoperative pain, and functional recovery. The results showed that the treatment outcomes in the experimental group were significantly better than those in the control group. Specifically, perioperative parameters and inflammatory response markers in the experimental group were significantly better than those in the control group, with statistically significant differences (*P* < 0.05). The overall treatment response rate in the experimental group was significantly higher than that in the control group (*P* < 0.05). Meanwhile, the incidence of postoperative complications in the experimental group was lower than that in the control group, postoperative VAS pain scores and ODI functional scores were lower, and postoperative JOA functional scores were higher than those in the control group, with statistically significant differences (*P* < 0.05). In conclusion, PCLE-LIF appears to be a promising technique with better clinical outcomes in the treatment of degenerative lumbar spinal stenosis.

## Introduction

Lumbar spinal stenosis is a prevalent condition in spinal surgery, encompassing a range of clinical symptoms caused by factors such as osteophyte or fibrous tissue proliferation and hypertrophy [1]. This results in a reduction in the normal sagittal diameter of the spinal canal or neural foramina, leading to irritation or compression of the spinal nerve roots or cauda equina [2]. While congenital factors may contribute to spinal stenosis, it is more commonly associated with degenerative changes in the lumbar spine, making it more prevalent among the elderly population [3].

In recent years, with the intensification of global aging, the incidence of degenerative lumbar spinal stenosis has gradually increased, significantly impacting the quality of life for middle-aged and elderly individuals. However, there is currently no clear evidence indicating an ideal conservative treatment for this condition, and surgical intervention remains the primary approach for its management. Different surgical strategies yield varying therapeutic outcomes [4–6]. Studies have shown that PCLE-LIF and TLIF play a significant role in treating degenerative lumbar spinal stenosis. PCLE-LIF employs percutaneous endoscopic technology, entering the intervertebral space through minimally invasive channels, utilizing an endoscope for the procedure. Relatively, PCLE-LIF causes minimal trauma and holds the potential to positively impact patient care, accelerating postoperative recovery. Conversely, TLIF is an open surgery typically performed by creating an intervertebral foramen. TLIF surgery involves a larger incision, providing more visual and operational space, but recovery may take longer compared to PCLE-LIF. The specific effects and efficacy of these procedures need further confirmation through additional research [7, 8].

The objective of this study is to explore and compare the efficacy of PCLE-LIF and TLIF in treating degenerative lumbar spinal stenosis, aiming to identify the optimal treatment approach for this condition. By highlighting the prospective nature of PCLE-LIF and its positive impact on patient care, we aim to provide readers with a more comprehensive understanding and insights for future research directions.

## Materials and methods

### The selection of research object data

**Fig. 1:**
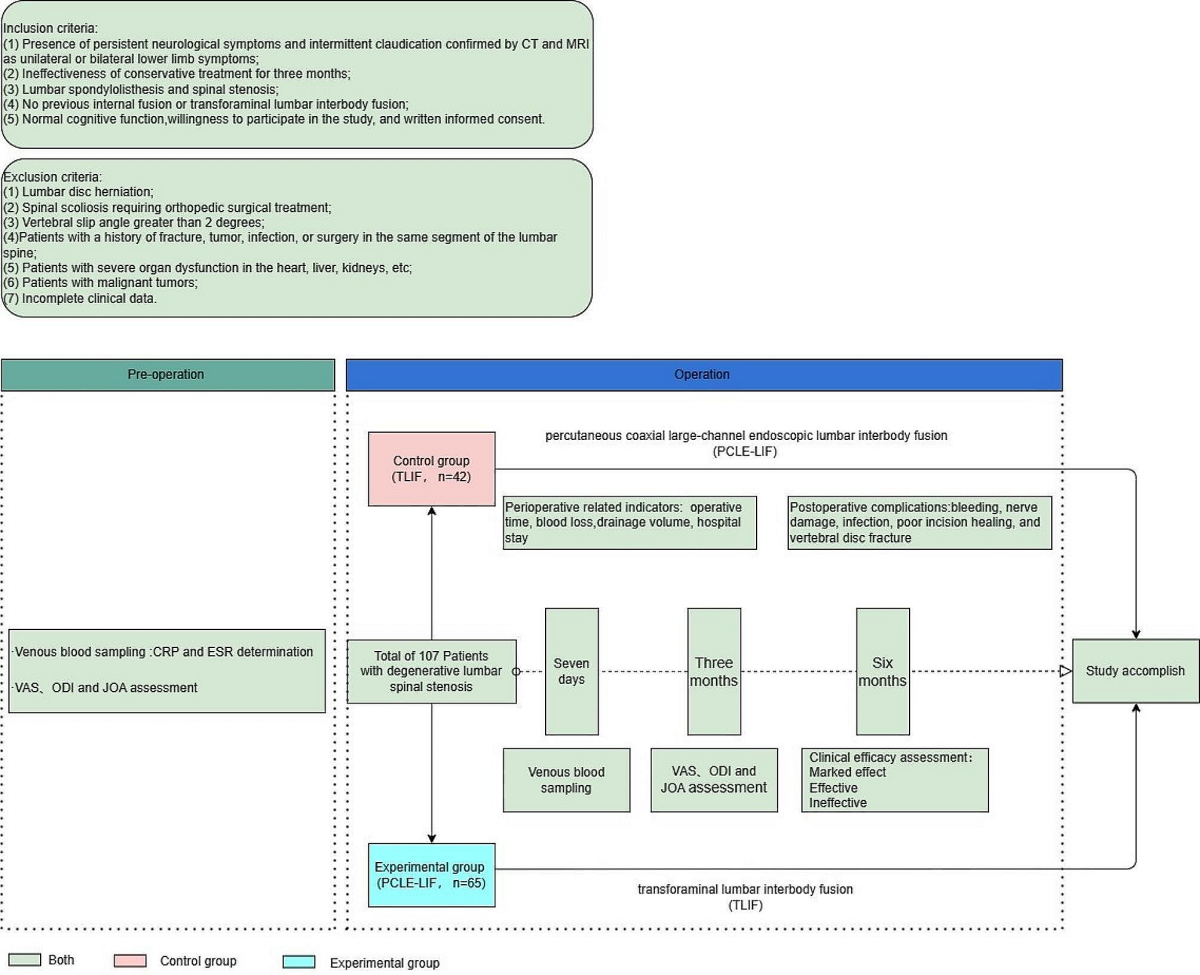
Flowchart of participant enrollment and study design

A retrospective study was conducted, involving 107 patients diagnosed with degenerative lumbar spinal stenosis who received treatment at the General Hospital of Ningxia Medical University and Ningxia Hui Autonomous Region People’s Hospital from September 2019 to September 2021. The patients were divided into a control group (42 cases) and an experimental group (65 cases). This study has obtained approval from the Ethics Review Committee of Ningxia Medical University (IRB No.: 2023-GJCG-001). The inclusion criteria of patients were: (1) Presence of persistent neurological symptoms and intermittent claudication confirmed by CT and MRI as unilateral or bilateral lower limb symptoms; (2) Ineffectiveness of conservative treatment for three months; (3) Lumbar spondylolisthesis and spinal stenosis; (4) No previous internal fusion or transforaminal lumbar interbody fusion; (5) Normal cognitive function, willingness to participate in the study, and written informed consent. The exclusion criteria of patients were: (1) Lumbar disc herniation; (2) Spinal scoliosis requiring orthopedic surgical treatment; (3) Vertebral slip angle greater than 2 degrees; (4) Patients with a history of fracture, tumor, infection, or surgery in the same segment of the lumbar spine; (5) Patients with severe organ dysfunction in the heart, liver, kidneys, etc.; (6) Patients with malignant tumors; (7) Incomplete clinical data. The experimental group underwent PCLE-LIF, while the control group received TLIF. **See** Fig. 1.

### Therapeutic method

The experimental group underwent PCLE-LIF. The specific procedure was as follows: Firstly, the condition was determined through lumbar spine CT and 2D reconstruction to provide detailed anatomical information for the surgery. After effective anesthesia and electrophysiological monitoring, the patient was placed in a prone position under general anesthesia. Routine disinfection and draping of the lower back were performed. Four bags of 3 L saline solution were hung on an adjustable stand at the head end, connected to both sides of the endoscope to increase water pressure. Surgical towels formed a dam around the operation area, and the operation field and surgical instrument placement area were covered in waterproof material to prevent leakage and ensure sterility. During the operation, a puncture was made at the marked site with a standard puncture needle, and C-arm fluoroscopy was used to confirm the surgical segment. The skin was incised with a sharp knife blade, and the pedicle puncture was performed using the pre-operative planned puncture route. After the puncture, a memory guide wire was placed, and the tail end of the guide wire was fixed on both sides of the operation area. Through the incision of implant nail, the myometrium was cut to 2 cm away from the central line of spinous process, and the muscle was passively separated and put into the step-by-step expansion tube and working channel. First, the soft tissue of the facet joint and the lower lamina margin were scraped under blind vision with a flat working channel, and the remaining soft tissue were removed with radiofrequency ablation electrodes and nucleus pulposus forceps to clearly expose the bony structure. Subsequently, a circular trephine or osteotome under the microscope was used to remove part of the inferior articular process. The upward resection range should reach the insertion point of ligamentum flavum, and the outward resection range should reach the upper articular process. Lamina rongeur or microscopical osteotome were used to gradually remove the upper articular process and caudal to the base of the upper articular process or the upper edge of the pedicle. The specific scope of bone structure resection is determined according to the operation space and decompression requirements. After the resection of the bony structure, the flat working channel was replaced by the oblique working channel to continue to complete the steps of intervertebral fusion. The long lingual surface of the oblique passage is used to protect the nerve, and the intervertebral space is treated under direct vision. The vertebral space is treated with lamina rongeur, reamer, scraper and curette, and the depth of the instrument into the intervertebral space is strictly limited. After intervertebral treatment, a trial model was placed into the intervertebral space to determine the size of the fusion cage. The bone grafting funnel is used to fill the intervertebral space with autologous bone particles. Then, the cage filled with autologous bone is implanted into the intervertebral space. The position of the cage is determined by C-arm fluoroscopy. The pedicle screw and connecting rod with appropriate length were implanted through the reserved track of memory guide wire, and the tail cap was placed and locked. After sufficient hemostasis, a drainage tube was placed and the wound was sutured layer by layer.

The control group underwent TLIF treatment. The brief procedure was as follows: Preoperative preparation was the same as that of the experimental group. After effective abdominal block anesthesia, the patient was placed in a prone position. A longitudinal midline incision was made, and using C-arm fluoroscopy, the affected segment was identified. Bilateral paravertebral muscles were excised, the costovertebral junction was located, and appropriate pedicle screws were inserted. A portion of the lower and upper articular processes on the surgical side was removed, and part of the yellow ligament was excised. Traction was applied to protect the nerve roots and dura mater, exposing the annulus fibrosus. The core of the intervertebral disc was removed, the cartilaginous plate was scraped off, and crushed autogenous bone particles were placed in the anterior one-third of the intervertebral space. An appropriately sized interbody fusion cage filled with bone chips was inserted. The contralateral articular surfaces and lamina were cleaned, and 360° fusion was completed using autogenous bone grafts. After confirming no nerve compression during exploration, the wound was irrigated, a negative pressure drainage tube was placed, and the incisions were closed layer by layer. The entire surgical process achieved interbody fusion by removing the lamina, cleaning the intervertebral disc, inserting interbody fusion material, and internal fixation, contributing to the stabilization of the spine and symptom relief.

### Observational index

Clinical efficacy, perioperative indicators, inflammatory response markers, postoperative pain, changes in lumbar function, and complications were observed and compared between the two groups.

(1) Perioperative related indicators: Mainly including operative time, blood loss, drainage volume, and hospital stay.

(2) Clinical efficacy: The treatment outcomes of the two groups were compared. A 6-month follow-up was conducted. The efficacy assessment was as follows: Marked effect: disappearance of lumbar and leg pain and swelling symptoms, significant joint function recovery, normal lumbar and leg muscle strength, and straight leg raising > 70°. Effective: improvement in lumbar and leg pain and swelling symptoms, relief of joint function, lumbar and leg muscle strength reaching level IV, and straight leg raising between 30° and 70°. Ineffective: no relief in lumbar and leg pain and swelling symptoms, no improvement or worsening of joint function, muscle strength at level I, and straight leg raising < 30°. The total effective rate = (Marked + Effective) / Total cases × 100%.

(3) Inflammatory response markers: In both groups, 10 ml of peripheral venous blood samples were collected in the early morning on an empty stomach before surgery, leaving 3 ml of blood. After centrifugation, the blood was allowed to stand for 10 min, and the serum was tested within 24 h. Enzyme-linked immunosorbent assay (ELISA) was used to detect C-reactive protein (CRP) and erythrocyte sedimentation rate (ESR). Assessments were conducted preoperatively and 7 days postoperatively to observe the impact of different fixation and fusion techniques on patients’ inflammatory response.

(4) Pain intensity: Visual Analog Scale (VAS) was used to assess the degree of lumbar and leg pain before and after surgery, with scores ranging from 0 to 10. Higher scores indicate more severe pain. VAS scale: 0–3 for no pain; 4–7 for pain; >7 for severe pain [9].

(5) Lumbar function: Oswestry Disability Index (ODI) scoring was used to evaluate lumbar function, including pain intensity, sleep disturbance, self-care, social life, walking, lifting, standing, sitting/lying, sexual activity, and travel. Each question had 6 options, with scores ranging from 0 to 5 points, totaling 50 points. The higher the ODI score after treatment, the more severe the lumbar functional impairment [10].

(6) Japanese Orthopedic Association (JOA) score includes scores for lumbar pain, leg pain and/or numbness, gait, straight leg raising test, sensory impairment, motor impairment, and bladder function, with scores ranging from 29 to 0. Lower scores indicate more severe functional impairment [11].

(7) Postoperative complications: Based on clinical observations of enrolled cases, perioperative complications may include bleeding, nerve damage, infection, poor incision healing, and vertebral disc fracture.

### Statistical analysis

The clinical information of patients in this study was gathered through our HIS electronic medical record system and ward physical examinations. Data collection was carried out under the guidance and supervision of clinical and medical record room doctors, utilizing standardized methods. On the day of data collection, a dedicated individual was responsible for verifying the database and promptly supplementing and correcting it. All clinical data were analyzed using SPSS 25.0 statistical software. Measurement data with a normal distribution are presented as means ± standard deviation, measurement data without a normal distribution are expressed as median and quartile spacing [M (P25-P75)], and inter-group comparisons were performed using independent *t*-tests or nonparametric tests. Counting data are expressed as percentages (%), and the χ2 test was utilized for comparisons of count data between groups. The significance level was set at α = 0.05.

## Result

### Demographic baseline characteristics

In the control group, there were 27 male and 15 female patients, aged between 47 and 78 years, with an average age of 63.67 ± 5.27 years. The research group consisted of 30 male and 35 female patients, aged between 49 and 76 years old, with an average age of 65.14 ± 6.10 years. There were no statistically significant differences in baseline data between the two groups (*P* > 0.05), ensuring comparability. See Table 1.

**Table 1:** Comparison of baseline data between the two groups

Group	*n*	Gender		Age (year)	Hight (cm)	Weight (kg)	BMI (kg/m^2^)	Surgical Segment
		Male	Female					L_4_-L_5_ L_5_-S_1_
Control group	42	27	15	63.67 ± 5.27	160.67 ± 9.71	64.15 ± 10.36	25.73 ± 3.70	25 17
Experimental group	65	30	35	65.14 ± 6.10	162.98 ± 7.67	67.42 ± 11.14	26.79 ± 3.35	34 31
t /χ²		1.320		1.282	1.368	0.410	1.533	0.537
*P*		0.251		0.202	0.174	0.682	0.128	0.552

### Comparison of perioperative monitoring indexes between the two groups

The perioperative monitoring indicators of the experimental group, including the time of operation (*P* = 0.001), amount of bleeding (*P* < 0.001), volume of drainage (*P* = 0.003), and hospital stays (*P* = 0.010), were all superior to those of the control group. See Table 2.

**Table 2:** Comparison of perioperative monitoring indexes between the two groups

Groups	cases(*n*)	time of operation (min)	amount of bleeding (ml)	volume of drainage (ml)	hospital stays (day)
Control group	42	147.32 ± 26.53	122.37 ± 17.35	78.63 ± 14.27	9.37 ± 2.94
Experimental group	65	126.47 ± 15.67	79.62 ± 14.57	57.18 ± 9.21	7.78 ± 2.45
*t*		3.637	7.629	3.151	3.915
*P*		0.001	< 0.001	0.003	0.010

### Comparison of clinical efficacy between the two groups

The total effective rate of the experimental group was significantly higher than that of the control group, and the difference between the two was statistically significant (*P* = 0.016). See Table 3.

**Table 3:** Comparison of clinical efficacy between the two groups

Groups	cases(*n*)	marked(*n*,%)	effective(*n*,%)	ineffective(*n*,%)	total effective rate(*n*,%)
Control group	42	16(39.10)	11(26.19)	15(35.71)	27(64.29)
Experimental group	65	34(52.31)	23(35.38)	8(12.31)	57(87.69)
*χ*2				8.285	
*P*				0.016	

### Comparison of inflammatory response indexes between the two groups

The ESR (*P* = 0.704) and CRP (*P* = 0.224) values of the two groups showed no statistical significance before treatment. However, after treatment, both ESR (*P* < 0.001) and CRP (*P* = 0.008) values in the experimental group were significantly better than those in the control group, and the difference between the two groups was statistically significant. See Table 4.

**Table 4:** Comparison of inflammatory response indexes between the two groups

Groups	cases(*n*)	ESR (mm/h)		CRP (mg/L)	
		Before	After	Before	After
Control group	42	16.91 ± 1.72	39.78 ± 4.52	7.52 ± 2.62	45.18 ± 3.75
Experimental group	65	16.68 ± 1.49	32.34 ± 4.18	7.65 ± 2.58	37.24 ± 3.48
*t*		0.380	7.629	1.023	3.642
*P*		0.704	< 0.001	0.224	0.008

### Comparison of pain and lumbar function scores before and after surgery between the two groups

The VAS pain score and ODI function score of the experimental group were lower than those of the control group, while the JOA score of the experimental group was higher, and the difference between the two was statistically significant (*P* all < 0.05). See Table 5.

**Table 5:** Comparison of pain and lumbar function scores before and after surgery between the two groups

Groups	cases(*n*)	VAS			ODI			JOA		
		pre-operation	three months	six months	pre-operation	three months	six months	pre-operation	Three months	six months
Control group	42	2.93 ± 0.62	4.52 ± 1.34*	4.12 ± 0.87*	46.65 ± 5.78	41.67 ± 6.18*	34.79 ± 5.79*	24.07 ± 1.53	9.40 ± 1.45*	2.20 ± 0.68*
Experimental group	65	3.05 ± 0.41	4.25 ± 1.23*	3.31 ± 0.64*	47.33 ± 5.32	35.73 ± 4.72*	28.74 ± 4.47*	24.07 ± 1.53	6.53 ± 1.41*	6.53 ± 1.30*
*t*		0.863	4.079	7.577	0.612	3.412	9.416	0.357	5.486	11.440
*P*		0.390	0.000	0.000	0.545	0.016	0.000	0.724	0.000	0.000

Note: * represents *P* < 0.05 when comparing data from the same group

### Comparison of complication rate between the two groups

In the experimental group, 4 patients experienced hemorrhage, 3 had nerve injury, 3 had infections, 2 had poor incision healing, and 1 had nucleus pulposus reprotrusion, resulting in a complication rate of 20.00% (13/65). In the control group, 8 patients had hemorrhage, 5 had nerve injury, 4 had infections, 4 had poor incision healing, and 2 had nucleus pulposus reprotrusion, leading to a complication rate of 54.76% (23/42). The complication rate in the experimental group was significantly lower than that in the control group, and the difference between the two groups was statistically significant. See Table 6. We believe that PCLE-LIF is generally considered a relatively minimally invasive surgical approach, which may result in fewer surgical traumas and a quicker recovery. This may contribute to reducing the risk of complications. However, individual patient differences, such as immune status and chronic diseases, could also influence the occurrence of complications. These factors should not be overlooked.

**Table 6:** Comparison of complication rate between the two groups

Groups	cases(*n*)	bleeding(*n*,%)	nerve injury(*n*,%)	infection(*n*,%)	poor wound healing(*n*,%)	nucleus pulposus re-protrusion(*n*,%)	no complication	overall complication rate(*n*,%)	χ2	*P*
Control group	42	8(19.05)	5(11.90)	4(9.52)	4(9.52)	2(4.76)	19(45.24)	23(54.76)	14.018	0.015
Experimental group	65	4(6.00)	3(4.61)	3(4.61)	2(3.08)	1(1.54)	52(80.00)	13(20.00)		

## Discussion

Degenerative lumbar spinal stenosis primarily refers to a reduction in the effective volume of the lumbar spinal canal due to abnormal bone or fibrous tissue components, leading to compression or irritation of neural tissue within the canal, resulting in functional impairment and a series of symptoms [12]. This is a complex, multifactorial disease. With the continuous aging of society, its incidence is on the rise, causing significant distress to patients and imposing a substantial burden on society [13, 14]. In cases where conservative treatment is ineffective, active surgical intervention should be considered. There are various surgical options for treating degenerative lumbar spinal stenosis, but regardless of the type of surgery, adequate decompression remains the key to symptom relief. Spinal surgeons can flexibly choose surgical approaches between minimally invasive or open procedures, fusion, and internal fixation, depending on the patient and institutional context, to achieve optimal treatment outcomes [15–17]. This study aims to explore the precise efficacy of PCLE-LIF and TLIF in the treatment of degenerative lumbar spinal stenosis, providing the best treatment options for patients with degenerative lumbar spinal stenosis.

The study found that PCLE-LIF play a significant role in the treatment of degenerative lumbar spinal stenosis, achieving higher clinical effectiveness, optimizing treatment outcomes, and promoting the recovery of lumbar function [18–21]. The study by Chang et al. showed that the intraoperative blood loss and postoperative drainage volume of PCLE-LIF were lower than those of the TLIF group [22]. The study by Liu et al. is consistent with the results of this study [23]. It may be because the PCLE-LIF procedure uses water as the medium. Under the action of water pressure, it can pre-stop bleeding of small blood vessels and clearly expose the operating field of view. At the same time, combined with radiofrequency ablation electrodes, can achieve more precise and rapid hemostasis. Joseph et al. reported that the incidence rates of sensory deficit, temporary neurological deficit and permanent neurological deficit in TLIF were 20.16%, 2.22% and 1.01% respectively [24]. A Mate analysis by Wu et al. showed that the incidence of nerve injury and dural injury in PCLE-LIF ranges from 3.3 to 10%. In this study, the incidence of nerve injury in TLIF group and PCLE-LIF group was 11.90% and 4.61% respectively [25]. This may be related to the long preoperative medical history and severe symptoms in some patients. In daily clinical practice, health education should be emphasized to encourage patients to seek early detection and treatment.

In this study, the experimental group and the control group underwent PCLE-LIF and TLIF, respectively. The results showed that, compared to the control group, the experimental group had better treatment outcomes, specifically characterized by a higher total effective rate, lower postoperative complication rate, lower postoperative VAS pain scores, and ODI functional scores, higher postoperative JOA functional scores, and better perioperative monitoring indicators, with statistically significant differences. The conclusions of this study are highly consistent with previous research, indicating the effectiveness of both surgical methods in treating degenerative lumbar spinal stenosis. However, in terms of efficacy and safety, PCLE-LIF demonstrated superior treatment performance. This may be attributed to the fact that PCLE-LIF represent important advancements in minimally invasive spinal surgery. Compared to other surgical approaches, traditional posterior spinal surgery can avoid drawbacks such as large incisions, more muscle cutting, increased bleeding, significant damage to posterior stable structure, and slow recovery. It can combine various minimally invasive retractors for nerve root decompression, nucleus pulposus extraction, and interbody fusion surgery, offering advantages such as minimal damage, less bleeding, short postoperative pain, quick recovery, shorter hospital stays, and better prognosis. In recent years, with the development of internal fixation devices and imaging techniques, the application of large-channel fusion and internal fixation under percutaneous endoscopy has become more refined, demonstrating clear therapeutic value and high applicability for patients with degenerative lumbar spinal stenosis [26–29].

This study is retrospective research and has some limitations: (1) No blinding was conducted; both the research group and patients were aware of the study grouping. Due to the subjective factors in the observed indicators such as VAS scores and clinical efficacy, patients in the experimental group may give higher ratings influenced by psychological suggestions, introducing bias to the study. In subsequent research, we will attempt evaluation by an independent assessor unaware of the study grouping to mitigate the potential influence of psychological suggestions on the experimental group, increasing the objectivity of the assessment. (2) The follow-up time is relatively short, evaluating clinical efficacy and recovery only in the short time after surgery. Future research should extend the follow-up time to assess long-term efficacy and quality of life. (3) The sample size of this study is relatively small, and it is a single-center study, introducing potential bias to the research results. In subsequent studies, we still need to increase the sample size, strengthen cooperation with other units, and conduct large-sample, multicenter studies to further evaluate the clinical efficacy of PCLE-LIF in the treatment of degenerative lumbar spinal stenosis.

## Conclusion

In summary, PCLE-LIF demonstrates a scientifically effective and significantly beneficial outcome for the treatment of degenerative lumbar spinal stenosis compared to TLIF. This approach, characterized by minimal trauma, expedited recovery, and reliable surgical intervention, provides patients with a more sustainable and comprehensive treatment effect. These research findings not only contribute to healthcare providers devising more precise personalized treatment plans but also offer patients a broader array of treatment options, thereby enhancing the overall quality of clinical practice.

## Data Availability

The datasets generated and/or analysed during the current study are not publicly available due to limitations of ethical approval involving the patient data and anonymity but are available from the corresponding author on reasonable request.
